# Factors influencing initial implementation of an online community-based exercise intervention with adults living with HIV: a systems approach

**DOI:** 10.3389/fresc.2023.1176960

**Published:** 2023-07-20

**Authors:** T. Jiancaro, A. M. Bayoumi, F. Ibáñez-Carrasco, B. Torres, K. McDuff, D. A. Brown, S. Chan Carusone, A. Tang, M. Loutfy, S. Cobbing, K. K. O’Brien

**Affiliations:** ^1^ Department of Physical Therapy, Temerty Faculty of Medicine, University of Toronto, Toronto, ON, Canada; ^2^ Institute of Health Policy, Management and Evaluation (IHPME), Dalla Lana School of Public Health, University of Toronto, Toronto, ON, Canada; ^3^ MAP Centre, St. Michael’s Hospital, Toronto, ON, Canada; ^4^ Department of Medicine, Temerty Faculty of Medicine, University of Toronto, Toronto, ON, Canada; ^5^ Dalla Lana School of Public Health, University of Toronto, Toronto, ON, Canada; ^6^ Therapies Department, Chelsea and Westminster Hospital NHS Foundation Trust, London, United Kingdom; ^7^ McMaster Collaborative Centre for Health and Aging, McMaster University, Hamilton, ON, Canada; ^8^ School of Rehabilitation Science, McMaster University, Hamilton, ON, Canada; ^9^ Maple Leaf Medical Clinic, Toronto, ON, Canada; ^10^ The Institute of Education Research, University Health Network, Toronto, ON, Canada; ^11^ Department of Physiotherapy, University of KwaZulu-Natal, Durban, South Africa; ^12^ Rehabilitation Sciences Institute (RSI), University of Toronto, Toronto, ON, Canada

**Keywords:** cognitive work analysis, systems engineering, implementation science, hiv/aids, physical activity, exercise. factors influencing initial implementation

## Abstract

**Introduction:**

Online community-based exercise (CBE) is a digital health intervention and rehabilitation strategy that promotes health among people living with HIV. Our aim was to describe the factors influencing initial implementation of a pilot online CBE intervention with adults living with HIV using a systems approach, as recommended by implementation science specialists.

**Methods:**

We piloted the implementation of a 6-month online CBE intervention and 6-month independent exercise follow up, in partnership with the YMCA in Toronto, Canada. We recruited adults living with HIV who identified themselves as safe to engage in exercise. The intervention phase included personalized exercise sessions online with a personal trainer; exercise equipment; access to online exercise classes; and a wireless physical activity monitor. Two researchers documented implementation factors articulated by participants and the implementation team during early implementation, defined as recruitment, screening, equipment distribution, technology orientation, and baseline assessments. Data sources included communication with participants; daily team communication; weekly team discussions; and in-person meetings. We documented implementation factors in meeting minutes, recruitment screening notes, and email communication; and analyzed the data using a qualitative descriptive approach using a systems engineering method called Cognitive Work Analysis.

**Results:**

Thirty-three adults living with HIV enrolled in the study (*n* = 33; median age: 52 years; cis-men: 22, cis-women: 10, non-binary: 1). Fifty-five factors influencing implementation, spanned five layers: (i) *Natural*, including weather and the COVID-19 virus; (ii) *Societal*, including COVID-19 impacts (e.g. public transit health risks impacting equipment pick-ups); (iii) *Organizational*, including information dissemination (e.g. tech support) and logistics (e.g. scheduling); (iv) Personal, including physical setting (e.g. space) and digital setting (e.g. device access); and (v) *Human*, including health (e.g. episodic illness) and disposition (e.g. motivation). The implementation team experienced heightened needs to respond rapidly; sustain engagement; and provide training and support. Additional organizational factors included a committed fitness training and research team with skills spanning administration and logistics, participant engagement, technology training, physical therapy, and research ethics.

**Conclusion:**

Fifty-five factors spanning multiple layers illustrate the complexities of online CBE with adults living with HIV. Initial implementation required a dedicated, rehabilitation-centred, multi-skilled, multi-stakeholder team to address a diverse set of factors.

## Introduction

Physical activity and exercise are rehabilitation strategies that benefit medically stable individuals with HIV (1). One model involves Community-based Exercise (CBE). CBE features exercise interventions designed by accredited professionals to boost regular physical activity for those in the community (2–4). CBE is traditionally delivered in-person.

Interest in telerehabilitation (5) and other digital health technologies is increasing. Stakeholders in the HIV community, for instance, have expressed interest in CBE that is online (6), while rehabilitation researchers have continued to explore new devices, such as physical activity monitors that are “smart” (7). These technological explorations are occurring against a backdrop of already existing challenges regarding uptake of physical activity. Even in the absence of new technologies, physical activity amongst people with HIV is variable, influenced by “a range of complex factors” (8). In the presence of new technologies, such as online CBE and wireless physical activity monitors, the implications for implementation are even less clear.

A key implementation question that behaviour change researchers face as they study new and modified digital health interventions is, “What works for whom in what settings to change what behaviors, and how?” (9). Digital health interventions include online rehabilitation services that use information and communication technologies such as wearable devices, interactive websites, and videoconferencing software (9). In 2017, implementation science specialists presented recommendations to develop and evaluate digital health interventions, which included recognizing the complexity of digital health behaviour change; adopting a “transdisciplinary” outlook; and considering approaches from “systems engineering” and “systems science” (9).

A system is defined variously as a “group or set of related or associated things…thought of as a unity”, which may include “persons working together as parts of an interconnecting network”; “artificial objects organized for a particular purpose”; and “natural objects…forming a connected or complex whole” (10). However, despite the 2017 recommendations (9), health researchers maintain that system complexity remains “much talked about but sub-optimally studied” (11). One problem involves ill-defined accounts of what an *intervention* is (12) and what its *context* is (13), while a related problem involves characterizing the connections between the two (13). Consequently, there is a continued push for more holistic approaches to health research, specifically ones that adopt complex systems thinking (2), described by the United Kingdom's Medical Research Council as, “focusing on the interactions between entities that comprise a system and between those entities and their environment, rather than assuming that a system can be understood by breaking it down into its individual entities and studying each part separately” (14).

Systems that are sociotechnical involve a mix of people, artifacts, and technologies (15). Digital health behaviour change interventions, such as those that use fitness trackers or apps, are sociotechnical in nature. For example, consider an intervention with adults living with HIV using exercise equipment and wireless physical activity monitors (WPAMs). This intervention functions within a constellation of contextual factors embedded within various environments or layers. These factors may include seasonal light levels (in a natural environment), external stigma (in a gym/social environment), device-app functionality (in a technological environment), and access to training shoes (in a personal environment). By understanding the various contextual factors that populate each environment or layer, researchers can better answer the key implementation question, “what works” (9).

Cognitive Work Analysis (CWA) is an engineering approach intended to study complex sociotechnical systems (15, 16). This approach offers researchers a conceptual modeling framework and analytical tools that zero in on the environmental factors that shape human behaviour. In CWA, complex sociotechnical systems include those with potentially numerous, “dynamic”, “diverse”, interconnected, and geographically dispersed components, with data that may be “uncertain”, and effects that may be “unanticipated” (15). In fact, CWA specifies 11 “characteristics” associated with sociotechnical complexity (see Methods). It also describes systems in terms of factors that can occur in different environments or, in CWA terminology, “layers” (see Methods). These layers are interwoven such that “All of the layers come together to shape the performance of the system as a whole” (15). Continuing with the WPAM example, the natural, social, technological, and personal layers must consequently come together for an adult living with HIV to produce a certain result. A holistic perspective that accounts for all of the layers is therefore crucial.

A CWA approach has been adopted for various healthcare applications (17), including health behaviour change studies that range from medication management to self-care management (e.g., 18–21). However, to our knowledge this approach has yet to be applied in the context of HIV.

In this article, we adopt a CWA perspective regarding an online community-based exercise (CBE) intervention among adults living with HIV. Collectively, this intervention and its context are regarded as the online CBE *system*. The purpose of this system is to improve or maintain health for adults living with HIV through exercise and physical activity.

Overall aims of this article involve describing and depicting an online CBE system; and identifying system factors influencing initial implementation. Specific objectives are as follows:

1. To describe and illustrate an online CBE system involving adults living with HIV in terms of (a) 11 characteristics of sociotechnical complexity and (b) 5 layers of sociotechnical complexity, in accordance with a CWA perspective; and
2. To identify factors that influence the initial implementation of an online CBE intervention with adults living with HIV, organized along the five CWA layers.

Initial implementation is important to study because obstacles in this phase can hamper full participation for some individuals, and thereby limit access. A systems approach may help researchers and clinicians better understand the context in which an intervention is situated and the conditions needed to support access.

## Methods

We used a CWA perspective (15, 16) to describe the factors influencing the early implementation of an online exercise intervention with adults living with HIV. The aim of the overarching Tele-coaching Exercise (TEx) Study is to pilot the implementation of an online community-based exercise intervention (22). In this systems-based sub-study, we specifically focused on the initial implementation phase of the intervention.

Activities associated with initial implementation included: (i) Participant recruitment, screening for eligibility, and consent to participate (Jul-Oct 2021); (ii) Exercise equipment distribution to participants (Oct-Nov 2021); and (iii) TEx Study orientation, and baseline fitness and questionnaire assessment (Oct-Dec 2021).

The TEx Study was approved by the University of Toronto Research Ethics Board (Protocol #40410). Evaluation of the TEx Study will be published elsewhere.

### Online community-based exercise intervention

**Figure 1:**
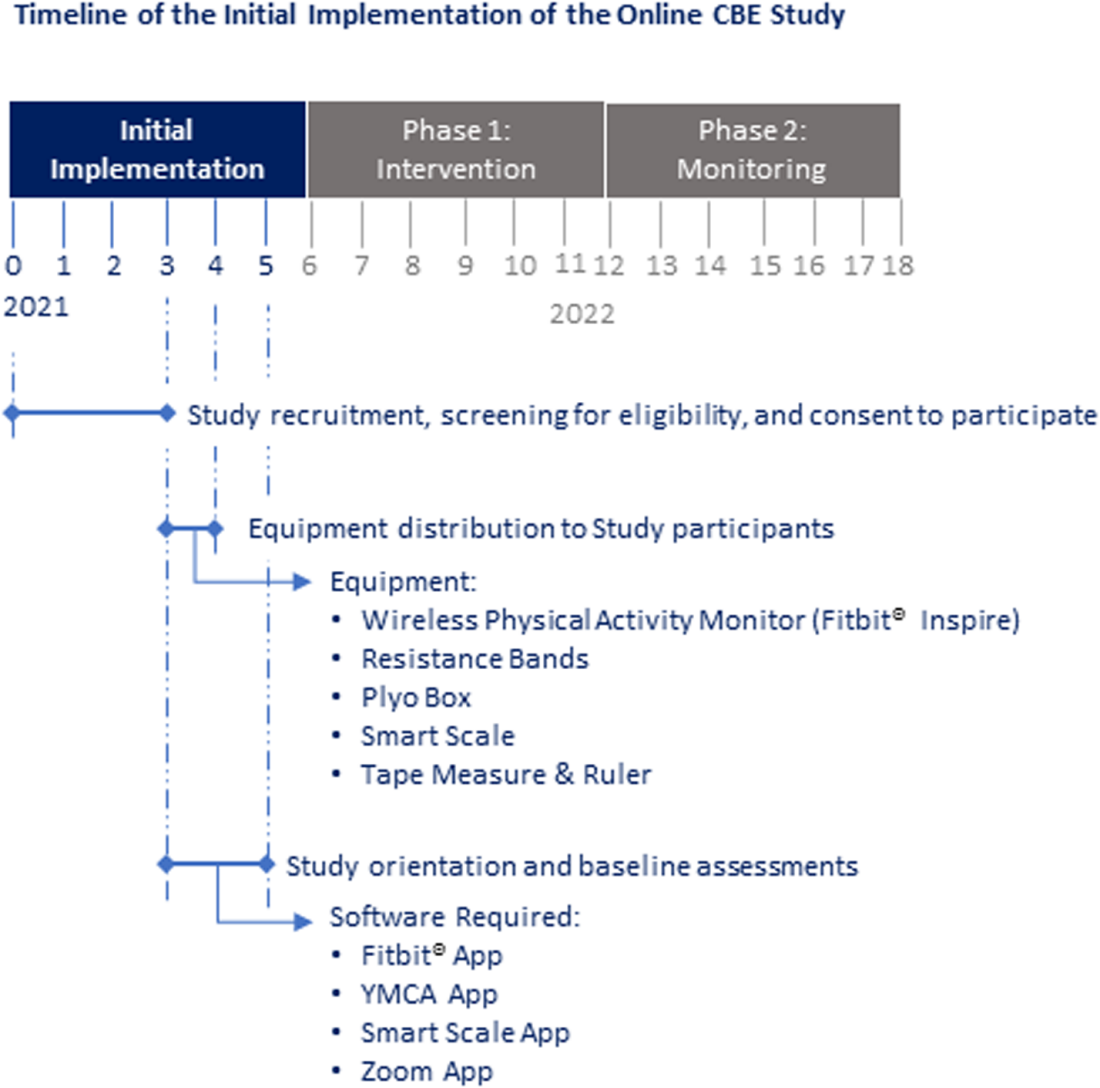
Overview of initial implementation of the online CBE intervention (details regarding phase 1 and phase 2 of the study are omitted for clarity).

The Tele-coaching Exercise (TEx) Study consists of a 6-month intervention phase and a 6-month follow-up phase, with adults living with HIV (Figure 1). The intervention phase included 13 bi-weekly personalized exercise sessions online with a personal trainer at the Toronto YMCA; 6 online group self-management educational sessions delivered monthly; home exercise and assessment equipment (resistance bands, a plyo box, and a smart scale); access to the YMCA online group exercise classes; and a wireless physical activity monitor (WPAM) to track physical activity (synced weekly), specifically the Fitbit® Inspire 2.

Personalized exercise sessions led by YMCA trainers involved aerobic, resistance, balance, and flexibility training (∼60 min, biweekly for 24 weeks). Type and intensity of exercise varied, depending on participants' abilities, goals and preferences. Dosage varied with participants' level of health, given the potentially fluctuating nature of their condition (23). Fitness and questionnaire assessments were administered online, at baseline and bi-monthly thereafter. The intervention included goal setting, exercise instruction, monitoring exercise progression, and feedback. Technology orientation involved an instructional handbook, electronic learning modules (e-modules), and one-on-one online tech support. Details regarding the data collection, assessments, and analysis for the TEx Study are available in the study protocol (22). See Figure 1 for an overview of the timeline of initial implementation.

#### Participants and recruitment

We recruited adults 18 years or older, living with HIV in Toronto, who considered themselves safe to participate in exercise as determined by the Physical Activity Readiness Questionnaire (PAR-Q) (24). To participate, individuals were required to have access to a device(s) (e.g., tablet, laptop or desktop computer, smartphone); access to Wi-Fi or a data internet plan; access to a webcam and willingness to activate it for group exercise classes, fitness sessions, assessments and educational sessions; access to a space in the home to exercise; and finally, willingness to participate in one year of the online exercise intervention, exercising thrice weekly in activities of their choice (∼60 min each time). We recruited participants via community-based organizations, via the Ontario HIV Treatment Network Cohort Study (25) at the Maple Leaf Medical Clinic, and via word of mouth.

#### Implementation team

The implementation team comprised fitness personnel and staff from the Central Toronto YMCA (responsible for personal training sessions, YMCA online platforms, and bi-monthly fitness assessments); and the University of Toronto research team (responsible for recruitment, screening, baseline questionnaire assessments, group self-management educational sessions, TEx Study orientation, equipment distribution at the YMCA, administration, budgeting, and research project management). The full team consisted of the following personnel:

• Central Toronto YMCA Core Fitness Personnel Team (5): 4 personal trainers, two of which performed bi-monthly online fitness assessments; and 1 Acting General Manager.
• University of Toronto Core Research Team (5): 2 TEx Study Co-Investigators, of which one functioned as an instructional designer, 1 Research Coordinator; 1 Post-doctoral Researcher; and 1 Engagement/Technology Coordinator.

Since initial implementation depended on the activities and interactions of these 10 team members, they were also considered participants of this sub-study, together with the TEx Study participants. In adopting a systems approach, we recognized that implementation team members comprised part of the overall system (16).

#### Initial implementation data sources

We examined the following data sources for this sub-study: research protocol; and documentation (notes) from (i) screening meetings for TEx Study eligibility with potential participants, (ii) research team meetings (held weekly over Zoom), (iii) meetings with the YMCA team and IT representative (held twice over Zoom), (iv) orientation sessions with the YMCA trainers (held twice over Zoom), (v) communication between participants, the research team and YMCA team (via email, Zoom and phone), and (vi) one time in-person meetings between the research team, YMCA staff and each participant during exercise equipment distribution at the Toronto YMCA.

### Cognitive work analysis approach

CWA comprises a perspective and tools to study mixed sociotechnical systems that are complex. This ecological perspective considers complexity in terms of various characteristics and layers (15).

Note that before applying formal CWA tools, a “knowledge elicitation” stage is required (15). This stage involves data collection using methods such as observation, interview, and document review. In this article, we describe knowledge elicitation using document reviews of the data sources (as listed above) pertaining to early implementation of the exercise intervention.

#### Eleven characteristics of sociotechnical complexity (objective 1a)

We applied the 11 characteristics of complex sociotechnical systems presented below (7) to the sub-study data sources to better understand the system in which the exercise intervention was embedded. The analysis was a subjective and pragmatic description of the system to identify components and their relations; and understand sources of complexity relevant to early implementation. The description was later reviewed by the research team. Note that the order of characteristics is not significant, and not all characteristics needed to be present for a system to be considered sociotechnically complex.

The 11 sociotechnical system characteristics are as follows: Large Problem Spaces (involving various “elements and forces”); Social (involving many individuals working in cooperation); Heterogeneous Perspectives (involving individuals with “potentially conflicting values”); Distributed (involving geographically distributed or dispersed individuals); Dynamic (involving changeable situations with potentially “long time constants” and delayed effects); Potentially High Hazards (involving negative effects on health, safety, finances or ecosystems); Many Coupled Subsystems (involving subsystems that are interconnected); Automated (involving automatic or algorithmic operations); Uncertain Data (involving “indicators” that may drift or fail); Mediated Interactions [involving “properties (that) cannot be directly observed by human perceptual systems”, such as intentions]; and Subject to Disturbances' (involving “unanticipated events”) (15).

#### System schematic (objective 1b)

**Figure 2:**
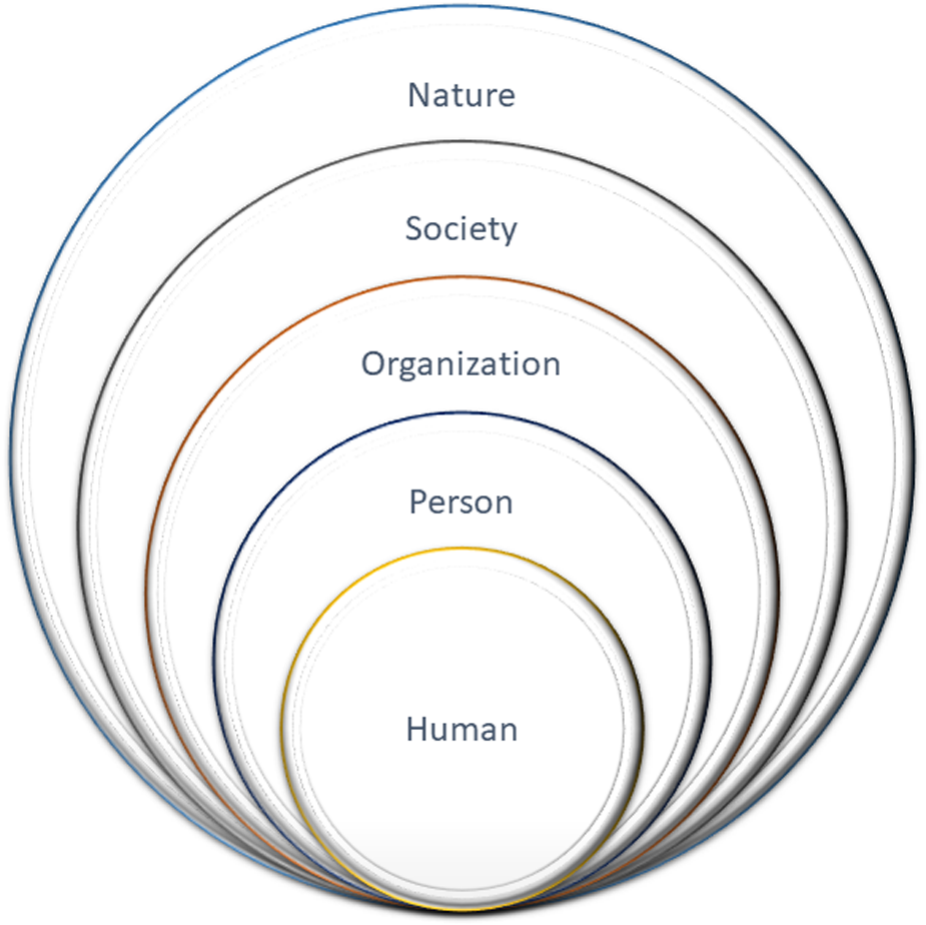
Five layers of sociotechnical complexity associated with an online community-based exercise system, adapted from vicente (15). Boundaries between layers are porous.

To complete this objective, a core team researcher and systems specialist (TJ), who also met with individuals to screen for eligibility to participate in the TEx Study, undertook an in-depth review of the sub-study documents (see Data Sources above), and drafted a system schematic for review by the research team. To generate the schematic, we used the review of the 11 characteristics listed above (Objective 1a) to help identify the system components; and the 5 layers of sociotechnical complexity (see Figure 2) to organize the components within the schematic.

The system schematic illustrates the primary components of the system (coloured in black) and helps set the scope for an ensuing analysis. Note that some components relevant to the system were consciously depicted outside the system boundary (coloured in grey) indicating they fell outside scope of the analysis. These secondary components included those most active outside the initial implementation period (e.g., funders) or components with which there was little to no interaction involving implementation team members (e.g., healthcare clinics, participant workplaces). Also depicted were entities that flow between primary components, such as goods, money, information, and personal data. These entities show some of the relations between primary components. The schematic was iteratively refined after the initial draft was reviewed by the full research team.

Regarding the layers, labelling them is system-specific, based on consensus, and intended to be flexible and pragmatic. To establish the system schematic for the CBE intervention, we defined layers as *human*, *person* (i.e., proximate social, physical, and digital settings), *organization(s)* including policies and procedures related to an intervention, *society* including customs and laws, and finally, *nature* (Figure 2). These layers are adapted from the CWA theory (15).

#### Initial implementation factors (objective 2)

The lead author identified the initial implementation factors (TJ) based on the data sources listed above. Once the factors were compiled, they were reviewed by the core research team, refined, and presented to the broader research team for feedback and verification. We used the five CWA layers to classify the implementation factors.

## Results

Forty-three individuals participated in the initial implementation period, of which 33 were adults living with HIV enrolled in the TEx Study (Table 1) and ten were members of the implementation team.

**Table 1:** Participant characteristics at study baseline in the online CBE intervention study (*n* = 33 participants).

Characteristic at study initiation	*N* (%)
Age, median	52 years
(Age range)	(33–71)
Gender	
Cis-Woman	10 (30%)
Cis-Man	22 (67%)
Non-binary	1 (3%)
Current marital or partnership status	
Single	12 (36%)
Married, common-law, partner or relationship	15 (46%)
Separated or Divorced	2 (6%)
Widowed	2 (6%)
Prefer not to answer	2 (6%)
Have children	9 (27%)
Live alone (*n* = 32)	13 (45%)
Average personal gross yearly income (CAD)	
Less than $30,000 CAD	15 (46%)
$30,000 to less than $60,000 CAD	10 (30%)
$60,000 to less than $100,000 CAD	6 (18%)
Greater than $100,000 CAD	2 (6%)
Main source of income	
Employment (full, part-time, or self)	12 (36%)
Income Support (e.g. Disability, Welfare, Worker's Compensation, Employment Insurance or Long Term Disability)	14 (42%)
Pension, Student Loans, or Savings	7 (21%)
Under the table work or Street-related work (e.g. pan-handling)	0 (0%)
Current employment status	
Employed (full time or part time)	15 (45%)
Student, Retired, or Volunteering	10 (30%)
Unemployed or on disability	8 (24%)
Highest level of education (*n* = 31)	
No formal education; secondary school completed	6 (19%)
Completed trade or technical training, or completed college	12 (39%)
Completed university or postgraduate education	13 (42%)
Race	
White	15 (46%)
Latin American, Hispanic or Latino (e.g. Mexican, Central/South American)	8 (24%)
Black or African American	8 (24%)
Asian (origins in far east, south east Asia, or Indian subcontinent including e.g. Cambodia, China, India, Japan, Korea, Malaysia, Pakistan, Philippine Islands, Thailand, Vietnam)	8 (24%)
First Nation (Indigenous), Inuit, Métis	3 (9%)
Prefer not to answer	1 (3%)
Year of HIV diagnosis	
Median (25–75th percentile)	2002 (1992–2012)
Undetectable viral load (<50 copies/ml)	30 (91%)
Concurrent health conditions (*conditions ≥30% of sample)*	
Cognitive decline (e.g. memory loss, confusion, trouble thinking clearly or solving day-to-day problems)	10 (30%)
Mental health condition	13 (39%)
Gastrointestinal conditions	16 (48%)
Trouble Sleeping	11 (33%)
Number of concurrent health conditions in addition to HIV	
2 or more	24 (73%)
Self-reported general health status	
Excellent	3 (9%)
Very good	12 (36%)
Good	10 (30%)
Fair	7 (21%)
Poor	1 (3%)
Health compared to previous year	
Much better	6 (18%)
Somewhat better	3 (9%)
About the same	15 (46%)
Somewhat worse	8 (24%)
Much worse	1 (3%)
Current state of exercise activity	
I currently do not exercise, do not intend to start	0
I currently do not exercise, but thinking of starting	8 (24%)
I currently exercise but not regularly	11 (33%)
I currently exercise regularly but only began in last 6 months	2 (6%)
I currently exercise regularly and have done so for >6 months	6 (18%)
I have exercised regularly in past but am not doing so currently	6 (18%)

Sixty-nine additional individuals communicated with the lead author (TJ) during recruitment and declined to participate. Of those 69 individuals, 18 expressed various concerns about the intervention and reasons for declining, which included the following: Lack of time/time commitment too high (*n* = 3); Lack of space (*n* = 2); Lack of motivation to exercise online (*n* = 2); Lack of privacy at home (re. disclosure) (*n* = 1); Not ready to commit to a one-year intervention (*n* = 1); Discomfort exercising in front of people (both in-person and online) (*n* = 1); Advised against participating by clinician (*n* = 1); Health reasons (*n* = 1); Technology issue (server problem) (*n* = 1); Lack of access to hardware (no webcam) (*n* = 1); Concerned about technological accessibility (*n* = 1); Has own personal trainer (*n* = 1); Belongs to a gym (*n* = 1); and Accepted employment out of town (*n* = 1).

### Sociotechnical characteristics of a complex online CBE system (objective 1a)

The description below refers to initial implementation of the online CBE intervention as it pertains to the 11 characteristics of a complex sociotechnical system.

#### Large problem space

The “many different elements and forces” as well as variables at play ranged from health situations to resource demands, technological challenges, and societal pressures (15). During screening interviews for eligibility and enrollment in the TEx Study, potential participants revealed a variety of health situations ranging from the impacts of HIV (e.g., fatigue, lipodystrophy) to the presence of comorbidities such as cardiovascular disease and depression and the impact of other health events, including a previous car accident and upcoming surgery. Potential participants raised other issues including access to technology (i.e., acquiring, using, and managing devices and peripheral hardware such as webcams), scheduling pressures (involving work and/or caregiving responsibilities), cost concerns (around internet data plans, future gyms memberships, and exercise equipment), pandemic restrictions (regarding gym closures), COVID-19 threats (considered “scary”), and finally, local weather conditions (specifically, wintry conditions).

#### Social

Of the 43 individuals involved in the initial implementation, 33 were participants who began the TEx Study at baseline. Ten individuals administered the intervention, working from two institutions (Central Toronto YMCA and University of Toronto). During initial implementation, we also planned self-management educational sessions, involving five subject matter experts who were invited to present various topics to participants online. Other stakeholders who met during initial implementation included the full research team, comprising 22 individuals operating from clinics, community-based organizations, and universities across the province and internationally (UK). Funding for the TEx Study (to implement the exercise intervention) came from a provincial organization.

#### Heterogeneous perspectives

Given the social nature of this intervention (see above), a variety of values, priorities and interests were conceivable. From potential participants, items raised during screening for eligibility to the TEx Study included privacy, motivation, and habit formation. From the implementation team, other concerns included intervention reach, budgeting, staff training, and TEx Study fidelity. Furthermore, the age range of participants was broad (between 33 and 71, see Table 1), contributing to the cohort's diversity of experience. Exercise histories prior to enrollment varied from little to no recent exercise to regular exercise (e.g., running, swimming). Consequently, participants expressed a range of exercise-related interests during the screening interviews (e.g., lose weight, improve strength, increase flexibility).

#### Distributed

As an online exercise study, the intervention involved participants who were geographically distributed across the Greater Toronto Area. Since initial implementation took place under public health restrictions during the COVID-19 pandemic, the intervention team all worked from their homes. Intervention delivery was administered via two separate institutions (the Central Toronto YMCA and the University of Toronto).

#### Dynamic

Health-related consequences of HIV are referred to as *episodic disability* and include physical, cognitive, and emotional symptoms as well as difficulties participating in social situations and performing daily activities of living (23). Consequently, we expected the health of some participants might fluctuate during the period between enrollment and baseline testing. One participant had surgery during this period. Other dynamic situations involved frequent technology software updates (re. WPAMs, Zoom, and personal devices) as well as shifts in participant work schedules, in pandemic restrictions, in COVID-19 viral mutations, and in seasonal conditions.

#### Potentially high hazards

With physical activity comes the risk of injury (mitigated by safeguards in keeping with YMCA policy). However, risks were not only physical. Mental health and other concurrent health conditions such as depression were raised by potential participants during screening for eligibility, and during subsequent baseline assessments.

#### Many coupled subsystems

Technically linked subsystems included device/internet interoperability (including synched WPAM accounts with YMCA accounts, and compatible hardware/software combinations to enable Zoom meetings). To enable communication amongst the implementation team, file sharing and encrypted servers were required. Other interacting subsystems involved procuring and assembling exercise equipment (requiring YMCA power tools); and managing and scheduling equipment distribution (involving either travel/transit for in-person pick-ups or home delivery work-arounds for those participants not travelling during the pandemic).

#### Automated

Several technology systems associated with the intervention functioned automatically (e.g., internet connectivity, app software updates, university server back-ups, and password management software).

#### Uncertain data

This characteristic was not a significant source of complexity during initial implementation. (Note: Data such as WPAM reports of “active zone minutes”, which count the duration of elevated heart rate activities, may be associated with uncertain data, but fell outside the initial phase of this sub-study.)

#### Mediated interaction

With the entire intervention administered online, every activity was mediated via computers, tablets, or smartphones, including communications over email and Zoom, WPAM orientation, baseline testing, data collection, project planning and administration, etc. Aspects of the intervention, such as the intentions and schedules of individuals, were not directly observable.

#### Subject to Disturbances

Disturbances came from a variety of sources between enrollment and intervention initiation, including episodic health disturbances (as outlined above), unexpected software updates, hardware problems, internet connectivity issues, videoconferencing glitches, and shifting pandemic-related risks and restrictions. There was a potential that widespread supply chain disruptions might delay equipment deliveries (of wood plyo boxes), and while there was a delay, it was not due to a supply chain problem (the driver could not find the drop-off location, requiring a re-order).

### Schematic of a complex online CBE system (objective 1b)

**Figure 3:**
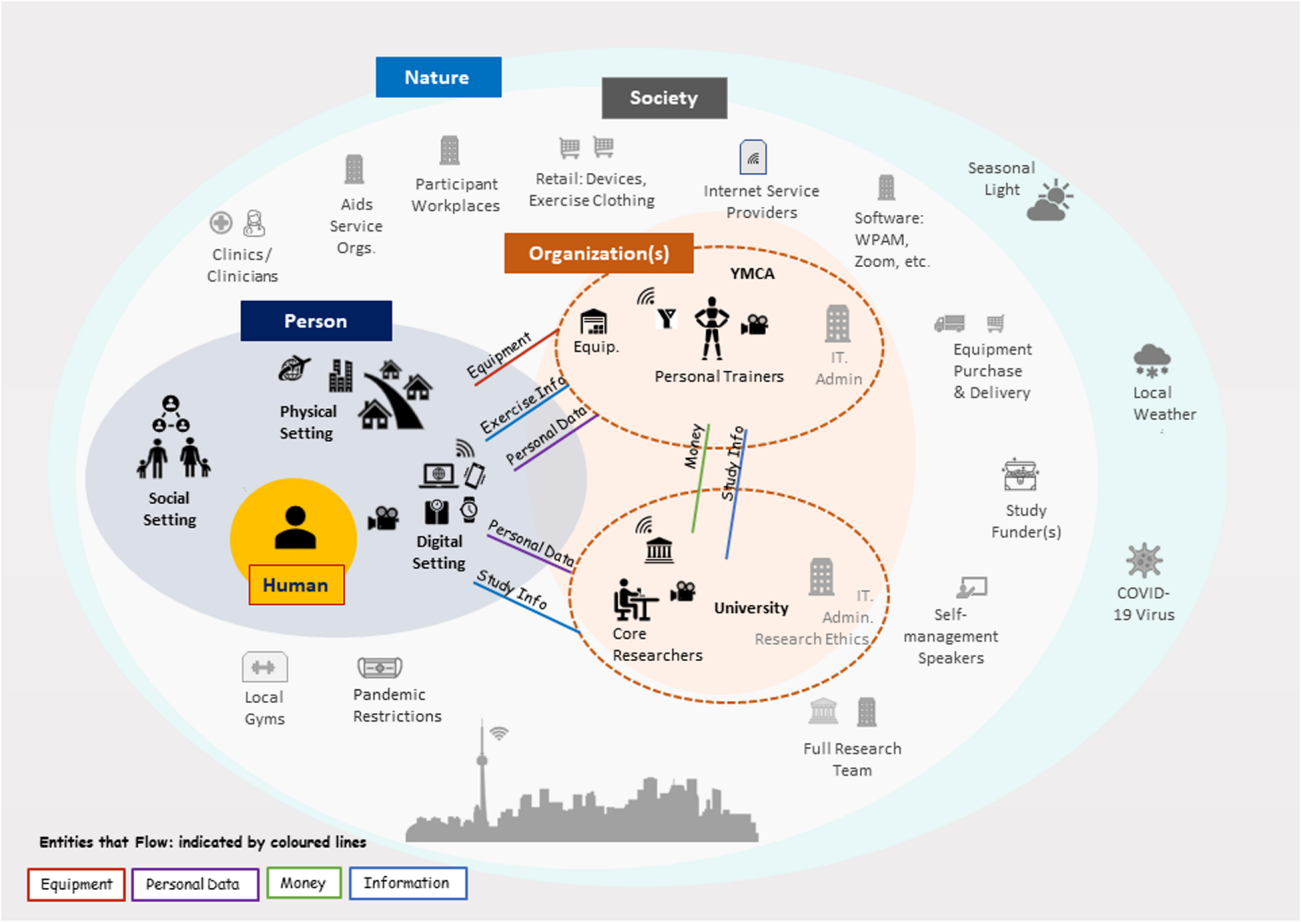
Schematic for the online exercise system. Components in black are primary to the system. (Toronto skyline by Bob Comix is licensed under CC BY 4.0).

The schematic (Figure 3) depicts components of the system according to the five layers of sociotechnical complexity introduced previously. Primary components (in black) are internal to the system under study and include participants and their physical, social, and digital settings, and staff at the two managing institutions (YMCA and University of Toronto). Secondary components (in grey) are external to the system and fall outside the scope of analysis. They include stakeholders such as the full research team, community-based organizations, healthcare clinics, and funders as well as technology suppliers (e.g WPAM outlets). Entities that flow between primary components are illustrated by coloured lines (i.e., money, personal data, TEx Study information, and exercise equipment). Entities flowing between primary and secondary components were omitted for clarity.

Regarding the characteristics of sociotechnical complexity (Objective 1a), they were illustrated in this schematic where possible. For example, many components are depicted, not only of the intervention, but also of the broader system at play (i.e., *large problem space*). For simplicity, the highly *social* nature of this intervention (43 individuals) is implied, though what is clear is the *distributed* nature of the intervention, with participants and team members at home (in their personal environments), two primary organizations (YMCA, University of Toronto) located in the city centre, other secondary organizations (e.g., health clinics) located throughout the city, and technology providers (e.g., WPAM manufacturer) in unknown locations. Interconnections between components span layers and were omitted for clarity. These include *automated* technical connections between devices (e.g., between a wearable WPAM and a phone app), social connections (e.g., between participants and team members), and societal connections (e.g., between public health restrictions and local gym closures).

### Initial implementation factors (objective 2)

**Figure 4:**
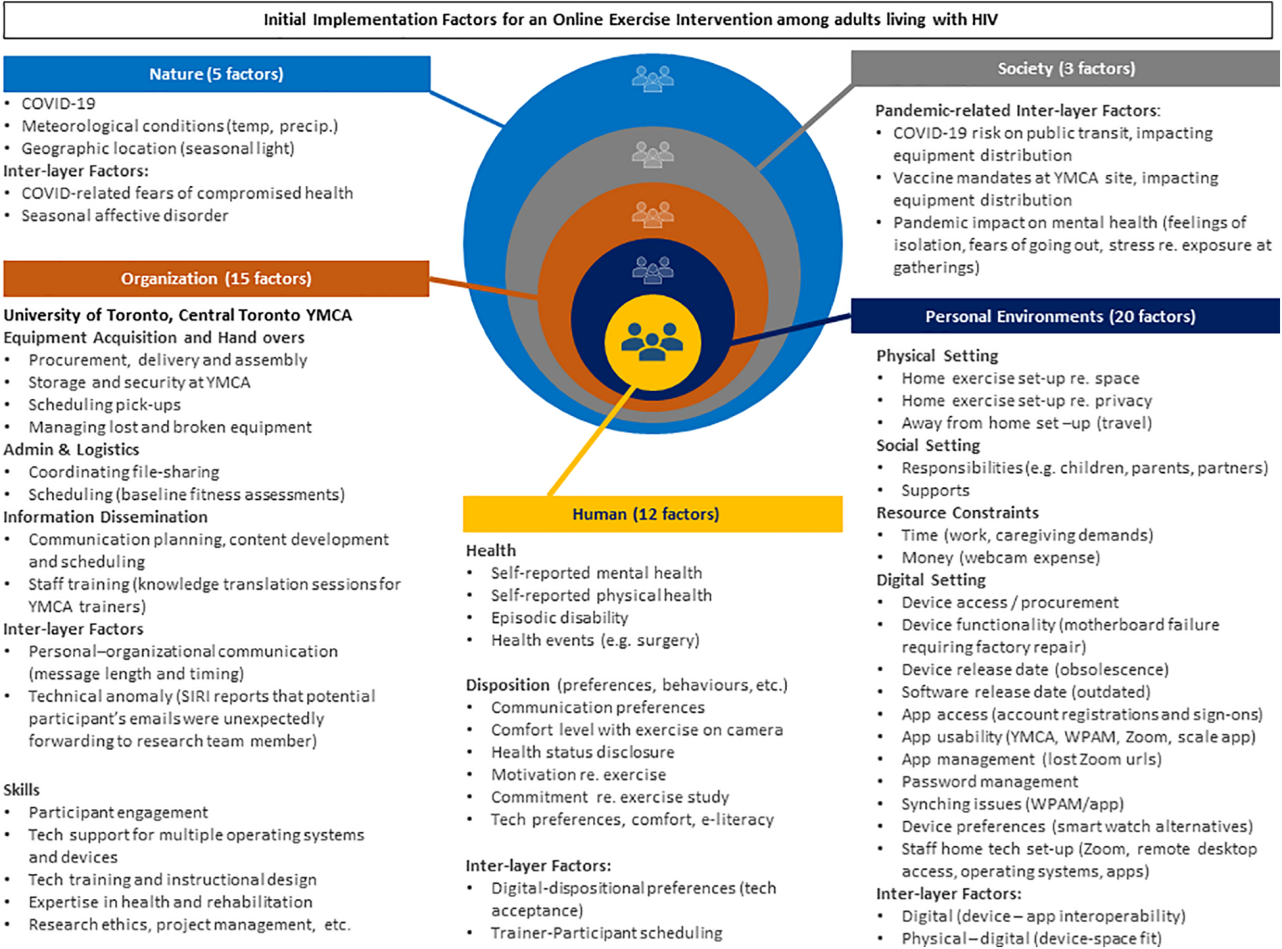
Fifty-five initial implementation factors that influence an online community-based exercise intervention with adults living with HIV.

We identified 55 factors influencing initial implementation (Figure 4), spanning five CWA layers, Natural, Societal, Organizational, Personal, and Human. The *Organizational* layer pertained to members of the implementation team only. All other layers pertain to both team members and potential participants. We describe the layers below.

#### Natural layer

Five factors arose in the natural layer during initial implementation. The presence of COVID-19 influenced virtually every facet of society, including personal layer fears of contracting COVID-19. Other factors raised by participants concerned worries of diminished activity levels in winter weather and seasonal affective disorder in low light conditions.

#### Societal layer

Three societal factors arose related to pandemic restrictions. Vaccine mandates at the YMCA and concerns over possible infection on public transit impacted the equipment distribution. Consequently, some participants requested home deliveries. To accommodate these requests, we applied for approval from the Research Ethics Board to obtain participant home addresses; and, to maintain privacy, one member of the team delivered the equipment throughout the city by car, rather than sharing addresses with a courier service. In addition, participants reported stress, and isolation due to the pandemic restrictions. Staff too were affected by COVID-19 pandemic restrictions (i.e., team members were under the same lockdown mandates and also susceptible to pandemic-related anxieties).

#### Organizational layer

Fifteen factors arose in the organizational layer, comprising the Toronto YMCA and University of Toronto. Substantial coordination was required to schedule in-person equipment distribution at the Toronto YMCA, which, prior to hand-over, also required the exercise equipment to be procured, assembled, stored, and delivered. Scheduling Zoom videoconferences for 33 participants to complete baseline questionnaires and fitness assessments also demanded significant coordination. Moreover, additional technology training was needed for several participants, requiring fast turn-around for technology orientation e-learning modules and phone/videoconference tech support.

#### Personal layer

Of the 20 factors in the immediate environment, 11 concerned technology, including password management, app usability, and device obsolescence. Other technology-related issues included a lack of access to a webcam, which was required for the TEx Study. Webcam set-ups proved challenging where physical space was limited, since trainers required a full-body view of participants as they exercised. Other factors related to social settings, including responsibilities that hampered exercise plans and supports that facilitated them. Resources constraints related to time to exercise and money to purchase equipment or memberships. Staff environmental issues included home videoconferencing set-ups that would ensure participant privacy.

#### Human layer

Twelve factors are listed in this layer. Regarding participants, several comorbidities were reported. Concurrent health conditions, including mental health, such as depression, were raised in screening interviews and during baseline assessments. Dispositional factors concerned aspects such as motivation to exercise and commitment to exercise thrice or more weekly. Inter-layer factors primarily concerned technology, for instance, participants reported that challenges setting up the technology caused stress.

Both participants and team members had communication preferences, some preferring phone to email, for example. Some individuals, including intervention team members, were new to Zoom videoconferencing and other technological aspects of the TEx Study, including WPAMs and online exercise platforms. This affected comfort with technology. Scheduling baseline fitness sessions also proved challenging for some at the outset, resulting in missed appointments. (Note that only the factors are reported here. Details of participant experiences will be reported in future.)

## Discussion

In this article, we adopted a systems engineering perspective regarding the initial implementation of an online exercise intervention study, involving 33 adults with HIV and 10 implementation team members. After reviewing 11 characteristics of complexity (Objective 1a), we devised a system schematic depicting the primary (internal to the system) and secondary (external to the system) components of an online exercise system (Objective 1b). Subsequently, we recorded 55 factors that arose during initial implementation, divided amongst five layers: *nature*, *society, organization*, *person*, and *human* (Objective 2).

In taking a CWA perspective, the complexity of this online exercise system became clear. With 43 individuals directly involved in the intervention, including both participants in the community and team members working from home, activity in this system was *distributed*, with behavior conditioned by organizational and societal norms, and influenced by natural conditions. Furthermore, with everyone's unique hardware-software configurations, there was a risk of significant technological challenges associated with *automated* events such as software updates, and *disruptive* events such as device failures. *Multiple interacting systems* made some events difficult to predict (e.g., Apple Siri reports that a potential participant's emails were unexpectedly forwarding to a research team member). The *large problem space*, populated by multiple variables, contributed to these events (15). Some TEx Study participants expressed reluctance to leave their webcams on, uncomfortable with exercising in front of others, possibly due to issues of body image, stigma, or privacy.

### Digital environments and issues of access

While digital rehabilitation interventions can be advantageous for some in terms of ease of “access”, “convenience”, and operating costs (26), there are disadvantages too. Technological issues, for example, dominated initial implementation of the online exercise intervention. Factors included the upfront costs of peripheral hardware, aging devices (such as smartphones), and e-literacy challenges (including managing app registrations and passwords). The difficulties stressed and frustrated some participants and required ongoing communication and tech support from the implementation team as well as rapid development of new instructional materials and e-learning modules. We learned that implementing a digital health intervention requires substantial investment of time and resources as well as a dedicated, multi-skilled team.

In the context of HIV, the technological difficulties we experienced may come as little surprise. A qualitative descriptive study exploring online exercise involving adults living with HIV described digital health as offering “geographical independence” for some, but also considerable challenges for others due to limited internet and device access (6). In low- and middle-income countries, for instance, investigators have identified outdated phones with limited memory capacities as a barrier to digital health for adults living with HIV (27). Although we implemented this intervention in a high-income country, we still found digital access to be a barrier for some (6). Consequently, we recommend that implementation specialists not only pay close attention to the minimum device requirements needed for app functionality, but also plan for technological challenges, including service outages, and software and hardware disruptions. In particular, we recommend developing training materials and, crucially, work-around procedures at the outset that anticipate technological failures.

### Complexity and HIV research

Researchers in implementation science are increasingly drawing on the perspectives and methods of complexity. The 2021 updated Medical Research Council's Framework for complex interventions now suggests addressing “sources of complexity”, listing entities associated with an intervention (e.g., “number of components”) and dimensions associated with a context (e.g., “social, political, economic and geographical”) (28). More technically, a computational approach within digital health models how “a participant's state can be represented in a multidimensional [contextual] state space” (29). The 55 system factors we uncovered, which range from the individual human layer to the natural world layer, are in keeping with these approaches, suggesting systems perspectives, like CWA, are becoming increasingly common within complex intervention and digital health research.

Likewise, in an HIV context, researchers continue to frame activities such as exercise in terms of its multiple layers. In one systematic review, the authors uncovered 55 possible “physical activity correlates”, divided into personal categories (i.e., “demographic”; “biological”; “behavioral”; and “psychological, cognitive and emotional” correlates), and environmental categories (i.e., “social/cultural”; “physical environment”; and “policy” correlates) (8). Similarly, investigators exploring considerations for engaging in online exercise with adults with HIV divided findings along “personal”, “structural”, and “community” dimensions (6). Our findings align with these categories and dimensions, and build on them by including aspects of the natural world and by considering how layers of a system may relate (by specifying inter-layer factors and identifying entities that flow between components and layers, like information).

Where a systems view appears to have taken root in the HIV research community is in considering social determinants of health. The World Health Organization defines these determinants as “the non-medical factors that influence health outcomes… the conditions in which people are born, grow, work, live, and age, and the wider set of forces and systems shaping the conditions of daily life. These forces and systems include economic policies and systems, development agendas, social norms, social policies and political systems” (30).

Researchers are beginning to investigate these determinants more deeply. Qualitatively, Safa et al. (2022), in their scoping review of physical activity involving people living with HIV, found gender, social support, social status and income to be among the most often studied in literature (31). While quantitatively, Hogan et al. (2021) explained how social determinants of health can be mathematically modelled with respect to HIV outcomes (32).

Theoretical and conceptual work can inform analytical models, and this is where approaches like CWA can contribute, by way of tools that help researchers understand the structure of a system. While we adopted a CWA perspective in this article and completed preliminary work associated with its knowledge elicitation stage (see Methods), we have yet to apply CWA's formal modeling tools. Future work involves developing a system map which presents a detailed description of *what* a system is, and *how* and *why* it functions (16). This multi-level means-ends map links objects and resources at a system's most concrete level (i.e., means) with overall purposes and values at its most abstract and intentional levels (i.e., ends). By proceeding with a hierarchical mapping, analysts can first conceptualize what a system is, including the context of an intervention; and then devise holistic evaluations that can help address the key implementation science question, “What works for whom in what settings to change what behaviors, and how?” (9). Without understanding the components that comprise a system, answering this question will remain problematic.

### Strengths and limitations

In this article, we adopted a systems approach to study the factors influencing initial implementation of an online exercise intervention in an HIV context. The strength of this approach is that it offers researchers a holistic perspective of a system, and conceptual tools to address complexity in a manner that respects the primacy of the environment and its impact on human behaviour (15). All rehabilitation systems may be studied in this light, which is akin to recognizing how environments, from the outset, can enable or disable.

Limitations relate to the flexibility of the CWA approach. Because a system in CWA can be parsed in many ways, no one way is considered correct. Much depends on the training and perspectives of the study team members and the purpose of the overall analysis. Regarding the system schematic, its aim is to illustrate a system pragmatically to further understanding, analysis, and stakeholder communication, so no formal rules governing the content or level of detail exist. Some depictions may benefit an analysis more than others. Other limitations relate to the 33 participants and 10 team members involved with the sub-study. A different set of individuals may have led to different results, including a different set of early implementation factors. Data sources were also subject to reporting inaccuracies, and their analyses, subject to misinterpretation. Furthermore, this sub-study was limited to initial implementation, disregarding factors that may be pertinent to other phases of the intervention. Finally, the sub-study took place in a Canadian urban centre, so how well these findings transfer to other geographical regions, particularly rural regions, is unclear.

## Conclusion

We identified 55 factors influencing initial implementation of an online exercise intervention for adults living with HIV that pertain to natural, societal, organizational, personal, and human layers. These factors illustrate the complexities of online exercise with adults living with HIV. Initial implementation required a dedicated, rehabilitation-centred, multi-skilled and multi-stakeholder team to address the diverse set of factors.

CWA can help guide the study of multi-component digital rehabilitation interventions for adults living with HIV, including how to incorporate social determinants of health and other environmental factors into a systems analysis. Future work includes systematically mapping factors influencing the full implementation to visualize and inform fidelity and broader online exercise scale-up in the context of HIV.

## Data Availability

The raw data supporting the conclusions of this article will be made available by the authors, without undue reservation.
